# Multi-Parameter Analysis of Biobanked Human Bone Marrow Stromal Cells Shows Little Influence for Donor Age and Mild Comorbidities on Phenotypic and Functional Properties

**DOI:** 10.3389/fimmu.2019.02474

**Published:** 2019-11-08

**Authors:** Anastazja Andrzejewska, Rusan Catar, Janosch Schoon, Taimoor Hasan Qazi, Frauke Andrea Sass, Dorit Jacobi, Antje Blankenstein, Simon Reinke, David Krüger, Mathias Streitz, Stephan Schlickeiser, Sarina Richter, Naima Souidi, Christien Beez, Julian Kamhieh-Milz, Ulrike Krüger, Tomasz Zemojtel, Karsten Jürchott, Dirk Strunk, Petra Reinke, Georg Duda, Guido Moll, Sven Geissler

**Affiliations:** ^1^BIH Center for Regenerative Therapies (BCRT), Charité Universitätsmedizin Berlin, Corporate Member of Freie Universität Berlin, Humboldt-Universität zu Berlin, Berlin Institute of Health (BIH), Berlin, Germany; ^2^Berlin-Brandenburg School for Regenerative Therapies, Charité Universitätsmedizin Berlin, Corporate Member of Freie Universität Berlin, Humboldt-Universität zu Berlin, BIH, Berlin, Germany; ^3^Julius Wolff Institute, Charité Universitätsmedizin Berlin, Corporate Member of Freie Universität Berlin, Humboldt-Universität zu Berlin, BIH, Berlin, Germany; ^4^Department of Nephrology and Internal Intensive Care Medicine, Charité Universitätsmedizin Berlin, Corporate Member of Freie Universität Berlin, Humboldt-Universität zu Berlin, BIH, Berlin, Germany; ^5^Institute of Medical Immunology, Charité Universitätsmedizin Berlin, Corporate Member of Freie Universität Berlin, Humboldt-Universität zu Berlin, BIH, Berlin, Germany; ^6^Department of Transfusion Medicine, Charité Universitätsmedizin Berlin, Corporate Member of Freie Universität Berlin, Humboldt-Universität zu Berlin, BIH, Berlin, Germany; ^7^BIH Core Unit Genomics Charité Universitätsmedizin Berlin, Corporate Member of Freie Universität Berlin, Humboldt-Universität zu Berlin, Berlin Institute of Health, Berlin, Germany; ^8^Berlin Center for Advanced Therapies, Charité Universitätsmedizin Berlin, Corporate Member of Freie Universität Berlin, Humboldt-Universität zu Berlin, BIH, Berlin, Germany; ^9^Spinal Cord Injury and Tissue Regeneration Center, Experimental and Clinical Cell Therapy Institute, Paracelsus Medical University, Salzburg, Austria

**Keywords:** cellular therapy, bone marrow stromal cell, mesenchymal stromal cell, *in vivo* and *in vitro* aging, comorbidity, *in vitro* potency assay

## Abstract

Heterogeneous populations of human bone marrow-derived stromal cells (BMSC) are among the most frequently tested cellular therapeutics for treating degenerative and immune disorders, which occur predominantly in the aging population. Currently, it is unclear whether advanced donor age and commonly associated comorbidities affect the properties of *ex vivo*-expanded BMSCs. Thus, we stratified cells from adult and elderly donors from our biobank (*n* = 10 and *n* = 13, mean age 38 and 72 years, respectively) and compared their phenotypic and functional performance, using multiple assays typically employed as minimal criteria for defining multipotent mesenchymal stromal cells (MSCs). We found that BMSCs from both cohorts meet the standard criteria for MSC, exhibiting similar morphology, growth kinetics, gene expression profiles, and pro-angiogenic and immunosuppressive potential and the capacity to differentiate toward adipogenic, chondrogenic, and osteogenic lineages. We found no substantial differences between cells from the adult and elderly cohorts. As positive controls, we studied the impact of *in vitro* aging and inflammatory cytokine stimulation. Both conditions clearly affected the cellular properties, independent of donor age. We conclude that *in vitro* aging rather than *in vivo* donor aging influences BMSC characteristics.

**Graphical Abstract:**
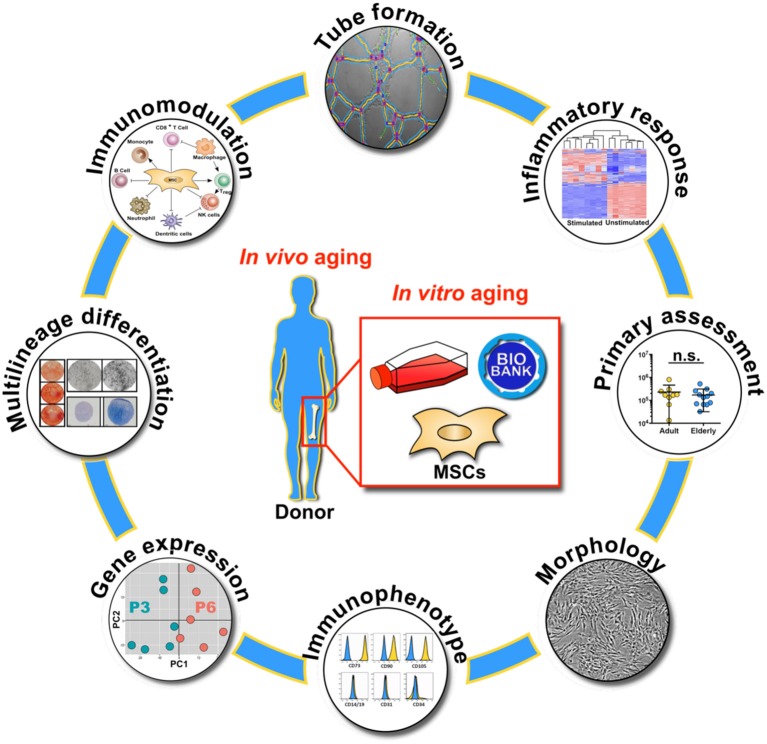
Overview on the molecular and functional assays used for the characterization of biobanked bone marrow stromal cells (BMSC) with respect to *in vivo* and *in vitro* aging, with primary assessment of starting material composition, cell morphology, immunophenotype, gene expression profile, multilineage differentiation capacity, immunomodulation, endothelial tube formation and inflammatory response.

## Introduction

Qualifying adult regenerative cell sources in biobanking approaches is an essential task in order to overcome major pitfalls in regenerative medicine (1). Donor-intrinsic variation between different cell batches may influence the safety and efficacy of bone-marrow stromal cells (BMSCs) (2–4). Our previous work suggests that multiple parameters, such as tissue origin (5–7), culture time (8, 9), media supplementation (7, 10), and mode of cell delivery (4, 9, 11–13) can substantially affect cellular therapeutic properties. In addition, advanced donor age and the commonly associated comorbidities are thought to be another substantial confounder of potentially compromising BMSC phenotype and function (14–22).

Previous studies investigating the impact of donor age on BMSCs reported variable or partly inconclusive outcomes considering their *in vivo* frequency, their gene expression profile, and many of their functional parameters, such as antioxidant defense, cytoskeleton dynamics, migration behavior, differentiation capacity, and immunomodulatory and paracrine activity (Table 1) (14–22). These discrepancies may result from differences in experimental parameters such as donor species, cell isolation, and culture protocols and from small sample size or limited functional characterization. Potential age-dependent impairments by chronological *in vivo* aging may be further aggravated by the process of *in vitro* aging during serial expansion in tissue culture (14, 16). Thus, the true impact of advanced donor age on the therapeutic value of BMSCs is still rather unclear.

**Table 1:** Literature study on *in vivo* and *in vitro* aging and/or comorbidities of mesenchymal stromal cells.

**Cell type (Tissue)**	**Type of aging**	**Model, donor source, donor number**	**Age range (Years)**	**Passage number**	**Parameters changed by aging and/or comorbidity**	**Ref**.
BMSCs	*In vivo*	Human; Healthy individuals; N = 8	16–32, 69–77	N.A.	(–) Proliferation (–) Immunophenotype (–) Metabolic activity (–) Trophic factor secretion *Therapeutic efficacy in C57/BL6 mouse model* ↓ Wound healing ↓ Neovascularization ↓ Trophic factor secretion ↓ Expression of genes involved in regeneration	(23)
BMSCs	*In vivo* *In vitro*	Human; Healthy individuals; N = 12	21–25, 44–55, 80–92	P2–P11	*In vivo* Altered gene expression (–) Cell size (–) Immunophenotype (–) Osteogenic, adipogenic, chondrogenic potential *In vitro* ↓ Adipogenic potential ↑ Osteogenic potential ↑ Senescence	(24)
BMSCs	*In vivo*	Human; Healthy individuals, Patients with hip arthroplasty; *N* = 16	≤ 23, ≥65	P1–P2	↓ CFU-F ↑ Senescence ↑ Cell size ↑ SASP- cytokine production (–) Cell viability (–) Immunophenotype	(25)
BMSCs	*In vivo*	Human; Patients with hip OA; *N* = 19	19–70	P2	↓ Proliferation ↓ Osteogenic potential ↑ Apoptosis	(26)
BMSCs	*In vivo* *In vitro*	Human; Healthy individuals; *N* = 25	2–13, 20–50	≤ P27	*In vivo* ↓ Proliferation (–) Immunophenotype (–) Telomere length *In vitro* ↓ Telomere length	(27)
BMSCs	*In vivo*	Human; Healthy individuals; *N* = 30	0–60	N.A.	↓ Proliferation ↓ Adipogenic potential ↑ Osteogenic potential Altered gene expression	(28)
BMSCs	*In vivo*	Human; Healthy individuals; *N* = 33	5–55	P1–P5	↓ CFU-F ↓ Proliferation ↓ Osteogenic potential ↓ Chondrogenic potential ↑ Cell size ↑ Apoptosis Altered immunophenotype (CD44, CD90, CD105, Stro-1) (–) Adipogenic potential	(29)
BMSCs	*In vivo*	Human; Healthy individuals; *N* = 36	41–86		↓ Proliferation	(30)
BMSCs	*In vivo*	Healthy individuals; *N* = 41	3–70	N.A.	↓ Number of osteoprogenitors	(31)
BMSCs	*In vivo*	Human; Healthy individuals; *N* = 46	≥18	P1–P3	(–) Cell number/sample weight (–) Immunophenotype (–) Proliferation (–) Osteogenic, adipogenic, chondrogenic potential	(32)
BMSCs	*In vivo*	Human Healthy individuals; *N* = 53	13–80	P1	Altered immunophenotype, (–) Proliferation (–) Adipogenic, osteogenic, chondrogenic potential (–) Immunomodulatory activity (–) Trophic factor secretion	(33)
BMSCs	*In vivo*	Human; Patients with cardiac complications; N.A.	1–5, 50–70	N.A.	↓ CFU-F ↓ Proliferation (–) Immunophenotype	(34)
BMSCs	*In vitro*	Human; Healthy individuals; *N* = 3	N.A.	P4, P8, P12	↓ Proliferation ↓ Immunomodulatory activity (–) Immunophenotype, telomere length (–) Metabolic activity	(35)
BMSCs	*In vitro*	Human; Healthy individuals; *N* = 6	20–40	P1–P9	↓ Proliferation ↑ Cell size ↑ Senescence ↑ Telomere length (–) Immunophenotype	(36)
BMSCs	*In vitro*	Human; Healthy individuals; *N* = 11	23–63	≤ P10	↓ Proliferation ↓ Adipogenic potential ↓ Osteogenic potential (–) Immunophenotype	(37)
BMSCs	*In vitro*	Human; Healthy individuals; *N* = 3	9,27,36	Early, late passage (≥ 38 PD)	Altered gene expression (–) Immunophenotype (–) Adipogenic and osteogenic potential	(38)
BMSCs	*In vivo*	Mouse; (C57Bl/6); *N* = 3	6 to 8-week-old ≥24-week-old	P2–P3	↓ Proliferation ↓ Osteogenic potential ↓ Immunomodulatory activity	(39)
BMSCs	*In vivo*	Mouse; (C57BL/6J); *N* = 6	3-month-old 16-month-old	N.A.	↓ Osteogenic potential ↑ Senescence ↑ Adipogenic potential	(40)
BMSCs	*In vivo*	Mouse; (C57BL/6); N.A.	6 to 8-month-old 20 to 26-month-old	N.A.	↓ Osteogenic potential ↑ Adipogenic potential	(41)
BMSCs	*In vivo*	Mouse; (SAMP6, SAMR1); N.A.	3 to 5-month-old	N.A.	↓ Osteogenic potential ↑ Adipogenic potential	(42)
BMSCs	*In vivo*	Mouse; (C57BL/6); N.A.	4 to 5-month-old 22 to 25-month-old	N.A.	↓ CFU-F	(43)
BMSCs	*In vivo* *In vitro*	Mouse; (C57Bl/6); *N* = 3	6 day-old 6 week-old 1-year-old	P1–P6	*In vivo* ↓ Proliferation ↓ Adipogenic potential ↓ Osteogenic potential ↓ Chondrogenic potential *In vitro* ↓ Adipogenic potential ↓ Osteogenic potential ↓ Chondrogenic potential	(44)
BMSCs	*In vivo* *In vitro*	Mouse; (BALB/c); *N* = 20	<4-week-old, 5 to 12-week-old 13 to 34-week-old	P3 - P24	*In vivo* ↓ CFU-F (–) Cell size (–) Proliferation (–) Immunophenotype (except CD73) (–) Adipogenic and osteogenic potential (–) Immunomodulatory activity *In vitro* ↓ Cell size ↑ CFU-F ↑ Proliferation ↑ Osteogenic potential (–) Immunophenotype (except Sca-1) (–) Adipogenic potential (–) Immunomodulatory activity,	(45)
BMSCs AT-MSCs	*In vivo*	Rat; (Lewis, Brown Norway); *N* = 12	4-week-old 15-month-old	N.A.	Altered immunophenotype (CD29, CD90, CD11, CD45)	(46)
BMSCs	*In vivo*	Rat; (Wistar); N.A.	12-month-old 24-month-old	N.A.	↓ Osteogenic potential	(47)
BMSCs	*In vivo* *In vitro*	Rat; (Sprague-Dawley); N.A.	3-week-old 12-month-old	≤ P100	*In vivo* ↓ Migration potential (–) Proliferation, osteogenic and adipogenic potential, cell size, *In vitro* ↓ Cell size ↓ Adipogenic potential ↓ Osteogenic potential ↓ Metabolic activity ↓ Gene expression involved in differentiation and mitochondrial functions (–) Proliferation	(48)
AT-MSCs	*In vitro*	Human; Healthy individuals; *N* = 3	N.A.	P5, P10, P15	↓ Proliferation ↑ Cell size ↑ Morphological heterogeneity (–) Osteogenic, adipogenic, chondrogenic potential (–) Immunophenotype (except CD105)	(49)
AT-MSCs	*In vivo*	Human; Healthy individuals; *N* = 8	0–1 70–80	P3–P8	↓ Proliferation ↓ Osteogenic potential ↓ Adipogenic potential ↑ Senescence	(50)
AT-MSCs	*In vivo*	Human; Healthy individuals; *N* = 24	6–12 22–27 60−73	P1–P5	↓ CFU-F ↓ Proliferation ↓ Osteogenic potential ↓ Adipogenic potential ↓ Migration potential ↑ Senescence (–) Cell viability (–) Immunophenotype	(51)
AT-MSCs	*In vivo*	Human; Patients with CAD & healthy individuals; *N* = 95	2–82	P2	↓ Angiogenic potential ↓ Telomerase activity (–) Immunophenotype;	(52)
AT-MSCs	*In vivo*	Human; Healthy individuals; *N* = 260	5–97	P0, P5	↓ Adipogenic potential (–) CFU-F (–) Proliferation (–) Osteogenic and chondrogenic potential	(53)
UC-MSCs	*In vitro*	Human; Healthy individuals; N.A.	>37 pregnancy week	P0–P16	Altered gene expression	(54)
**Cell type (Tissue)**	**Type of comorbidity**	**Model, donor source, donor number**	**Age range (Years)**	**Passage number**	**Parameters changed by comorbidity**	**Ref**.
BMSCs	T1D with renal failure	Human; T1D Patients & non-diabetic individuals; *N* = 31	18–70	P1–P5	Altered expression of genes involved in wound healing and stress response (–) CFU-F (–) Immunophenotype (–) Proliferation (–) Migration potential (–) Immunomodulatory activity	(55)
BMSCs	T1D	Human; T1D Patients and non-diabetic individuals; N.A.	23, 31	N.A.	(–) Cell size (–) Immunophenotype (–) Adipogenic differentiation (–) Immunomodulatory activity (–) Gene expression	(56)
BMSCs	DM,CLI, CAD	Human; Ischemic Patients (+DM) and healthy individuals *N* = 12	N.A.	P3–P6	↓ Proliferation in later passages (–) Immunophenotype (–) Angiogenic potential	(57)
AT-MSCs	T2D with CLI	Human; T2D Patients; N.A.	N.A.	N.A.	↓ Proliferation ↓ Migration potential ↓ CFU ↓ PDFG signaling ↓ Osteogenic potential ↑ Adipogenic potential ↑ Prothrombotic phenotype (–) Immunophenotype	(58–60)
AT-MSCs	T2D	Human; T2D and non-diabetic patients; *N* = 40	N.A.	N.A.	Altered immunophenotype (CD90, CD105) ↑ Expression of stemness markers (NANOG, OCT4) ↑ Oxidative stress ↑ Production of pro-inflammatory cytokines	(61)
AT-MSCs	T2D	Human; T2D and non-diabetic patients; *N* = 40	60–76	N.A.	↓ CFU ↑ Apoptosis ↑ Senescence (–) Proliferation	(62)
AT-MSCs	ATH T2D	Human; Patients with/without ATH; *N* = 50	<65 ≥65	P2–P3	↓ Immunomodulatory activity	(62)
AT-MSCs	Obesity T2D	Human; Healthy individuals, Patients with Obesity & T2D *N* = 12	30–55	P3–P7	↓ Immunomodulatory activity ↑ Metabolic activity ↑ Migration potential ↑ Expression of inflammatory markers	(63)
BM-ECs	T1D	Mouse; T1D and non-diabetic (CD1); N.A.	N.A.	N.A.	↓ Angiocrine activity, migration ↓ Angiogenic potential ↑ Transendothelial migration ↑ Permeability	(64)
BM-ECs	T1D	Mouse; T1D and non-diabetic (CD1); N.A.	N.A.	N.A.	↓ Hematopoietic fraction in bone ↓ Migration ↓ Angiogenic potential ↑ Osteopenia in bone ↑ Fat cells in bone ↑ Senescence ↑ Oxidative stress	(65)

*N.A., not available; **Cell types**: BMSCs, bone marrow stromal cells; MSC, mesenchymal stromal cells; **Tissue sources**: AT, adipose tissue; BM, bone marrow; UC, umbilical cord; P, passage number; **Parameters**: PD, population doubling; CFU-F, colony-forming unit fibroblast; SASP, senescence-associated secretory phenotype; PDGF, platelet-derived growth factor; **Comorbidities**: CLI, critical limb ischemia; T1D/T2D, type 1 and 2 diabetes mellitus; ATH, atherosclerosis; CAD, coronary artery disease; OA, osteoarthritis*.

We hypothesized that donor age, in combination with age-related comorbidities, contributes to the perceived large phenotypic and functional heterogeneity between individual donor-derived cellular specimens. Surprisingly, we found no substantial association between donor age or comorbidities and BMSC characteristics. In contrast, our analysis revealed that *in vitro* aging and inflammatory cytokine stimulation clearly alter cellular properties.

## Materials and Methods

### Isolation and Culture of Bone Marrow Stromal Cells (BMSCs)

BMSCs were received from the Core-Facility “Cell Harvesting” of the BIH Center for Regenerative Therapies (BCRT). The cells were isolated from metaphyseal bone marrow (BM) biopsies from patients undergoing hip replacement at Charité University Hospital, as previously stated (1, 66–68). Written informed consent was given, and ethics approval was obtained from the local ethics committee/institutional review board (IRB) of the Charité University Hospital.

Briefly, the BM mononuclear cell fraction (BM-MNC) in primary BM and the BMSC fraction post Ficoll-density gradient centrifugation (Histopaque 1077; Sigma-Aldrich) were quantified with an automated electrical impedance-based CASY® Cell Counter (Schaerfe System GmbH). The BMSC-containing interphase was plated in a 300 cm^2^ tissue culture flask (ThermoFischer) and cultured under standard conditions (37°C, 5% CO_2_) in an expansion medium (Dulbecco's Modified Eagle Medium-Low Glucose [DMEM-LG; Sigma-Aldrich] containing 10% fetal calf serum [FCS; Biochrom AG], 100 U/mL penicillin, and 100 μg/mL streptomycin [Biochrom AG], and 2 mM L-alanyl-L-glutamine [GlutaMAX; Gibco]). The non-adherent fraction was removed by washing with PBS (Gibco), the medium was changed every 72 h, and the cells were allowed to reach about 80% confluence before passaging.

The BMSCs were then expanded for several passages and were characterized with multiple functional and molecular assays, in line with the minimal criteria of the International Society for Cellular Therapy (ISCT) (69), at passage three (P3, early passage) and six (P6, late passage), respectively, as also shown in overview in the Graphical Abstract.

### Cell Morphology, Viability, Growth Kinetics, and Immunophenotyping

Cell morphology was determined at regular intervals by using bright field light microscopy. Cell number, viability, size, and volume were determined at each culture passage by using the CASY® Cell Counter as outlined previously (6, 9). BMSC growth kinetics were quantified by calculating population doublings at each passage based on the following equation: PD = log(N/N_0_)/log(2). In this formula, N stands for the total number of viable cells at harvest, and N_0_ is the initial number of cells seeded.

Flow cytometric immunophenotyping was conducted as described earlier (6, 9) using a BMSC Duraclone-panel (DURAClone SC Mesenchymal Tube; Beckman Coulter) containing the following antibodies: CD14, CD19, CD31, CD34, and CD45 (as negative markers) and CD73, CD90, CD105, and CD146 (as positive markers), or unlabeled control cells. Upon antibody labeling, the cells were washed with PBS, fixed with 1% paraformaldehyde, and analyzed on a Cytoflex flow cytometer (Beckman Coulter), and 5,000–10,000 gated events were quantified and analyzed with FlowJo v10.3.1 (FlowJo LLC).

### Gene Expression Analysis by RNA Sequencing

The mRNA transcripts of resting or cytokine-stimulated BMSCs were studied by seeding the cells at 2,000–4,000 cells/cm^2^ in 75 cm^2^ culture flasks and expanding them for 1 week. Before harvest, the sub-confluent cells were washed twice with PBS and lysed with 1 mL of RLT-buffer (Qiagen). Total RNA was extracted by using the Qiagen RNeasy Plus Mini Kit (Qiagen), according to the manufacturer's instructions. A total of *n* = 37 BMSC samples were analyzed (*n* = 24 samples for P3 and *n* = 6 for P6, which were matched to *n* = 6 of the P3 donors). A subset of *n* = 7 donors was treated for 24 h with or without cytokines (TNF-alpha and IFN-gamma, both 10 ng/mL), which were matched to the corresponding unstimulated cells and processed in parallel.

Total RNA was extracted by using the RNeasy Plus Mini Kit, and the quality was assessed by Bioanalyzer RNA 6000 Nano assay (Agilent). Only high-quality RNA with RIN scores > 7 was used for library preparations. The RNA (1 μg of total RNA) from each sample was converted to complementary DNA (cDNA) using an iScript^TM^ cDNA Synthesis Kit (Bio-Rad). Sequencing library preparation was performed using the NEBNext® Ultra^TM^ RNA Library Prep Kit for Illumina® and PolyA mRNA selected from 500 ng of total RNA with a NEBNextPoly(A) mRNA Magnetic Isolation Module (both New England Biolabs) followed by library preparation. Libraries were quantified with a Qubit® dsDNA HS Assay Kit (Thermo Fischer) and sequenced on a HiSeq 4000 System (Illumina) in single-read mode with a 50-cycle read length.

FASTQ-files were quality-controlled with “fastQC” and trimmed for residual adapter sequences and low-quality reads with “AdapterRemoval” (70). Reads were aligned to the GRCh38 human genome using “tophat” and “bowtie2” (71, 72). Counts per gene were calculated using the “featureCounts” algorithm implemented in the “Rsubread” package in R (73). Genes were annotated with the “biomaRt” package and Ensembl-Version 94. Protein-coding genes were selected, expression values normalized, and variance stabilizing transformed using the “DESeq2” package in R (74). Principle component analysis (PCA) was performed for the 1,000 genes with the highest variance across all samples. Differentially expressed genes between groups were determined using negative binomial distribution models as implemented in the “DESeq2” package. Raw *p*-values were adjusted for multiple testing with Bonferoni correction, and an adjusted *p*-value below 0.05 was used for the selection of significant genes. Functional annotation and enrichment analysis were carried out using “DAVID” with the “clusterProfiler” package in R (75). False discovery rates were used to adjust raw *p*-values for multiple testing, and a threshold of *p* < 0.05 was used for the selection of significant results. Dotplots of top-ranking results were created with the function implemented in the “clusterProfiler” package. GOcirc plots were created using the algorithm in the “GOplot” package in R (76). The raw data on expression are available at the Gene Expression Omnibus under the GEO-Accession-ID (GSE139073).

### Multilineage Differentiation Analysis

Adipogenic, osteogenic, and chondrogenic differentiation of BMSCs at P3 and P6 were induced by using specific differentiation media and evaluated as previously described (48, 66–68, 77–79). Briefly, BMSCs were plated in 24-well plates at specific densities for adipogenic (1.44 ×10^4^ cells/well) or osteogenic (1.28 ×10^4^ cells/well) differentiation or in V-bottom 96-well plates at higher density (3.0 ×10^5^ cells/well) for chondrogenic differentiation. Control cells were exposed to normal culture media, and all cultures were sustained for up to 22 days. To compare the differentiation potential of BMSCs among age groups and comorbidities and between passages, the differentiation responses from each individual were normalized to their respective controls.

#### Adipogenic Induction

BMSCs were cultured with complete DMEM-HG (High Glucose) supplemented with 10 μM dexamethasone, 50 μM indomethacin, 10 mM 3-isobutyl-1-methylxanthine, and 0.1 μM insulin (all from Sigma-Aldrich, St. Louis, MI, USA). Adipogenic differentiation was demonstrated by performing Nile Red staining (Sigma-Aldrich) to visualize lipid droplet formation. Quantification was achieved by measurement of Nile Red fluorescence (Ex/Em 485/540), which was normalized to the cell number quantified by staining with Hoechst 33258 dye (Life Technologies) and consecutive readout on a multimode microplate reader (TECAN M200 PRO) (67).

#### Osteogenic Induction

BMSCs were cultured with complete DMEM-LG supplemented with 0.1 μM dexamethasone, 50 μM ascorbic acid, and 10 mM beta-glycerol-phosphate disodium salt hydrate (all Sigma-Aldrich). Osteogenesis was assessed by Alizarin Red S (Merck) staining to determine mineralized matrix deposition, which was quantified at days 14, 18, and 22 by measuring the absorbance of Alizarin Red S and then normalized to the cell number determined by Hoechst staining, with consecutive readout of absorbance on the TECAN reader. The ALP activity level was quantified by measuring the consumption of p-nitrophenyl phosphate (pNPP; Sigma-Aldrich), which was normalized to the amount of viable cell metabolic activity as measured by PrestoBlue® assay (Life Technologies) (67, 78).

#### Chondrogenesis

BMSCs (only at passage 6) were placed in V-bottom 96-well plates, centrifuged and subsequently cultured for up to 21 days in a chondrogenic medium [FBS-free DMEM-HG supplemented with 6.25 μg/mL insulin-transferrin-selenium, 0.1 μM dexamethasone, 50 μg/mL L-Ascorbic acid 2-phosphate sesquimagnesium salt hydrate, 1 mM sodium pyruvate, 0.35 mM L-proline, 1.25 mg/mL bovine serum albumin, 5.35 mg/mL linoleic acid (all from Sigma-Aldrich), and 10 ng/mL transforming growth factor-beta-3 (TGF-beta; Peprotech)] (78), and to quantify chondrogenesis, proteoglycan production was normalized to total protein amount (67).

### Immunomodulation, Endothelial Tube Formation, and Cytokine Measurements

The immunomodulatory effects of BMSCs were assayed as described previously (80). Human peripheral blood mononuclear cells (PBMCs) were stained with 5 μM carboxy-fluorescein-succinimidyl ester (CFSE; Life Technologies), stimulated with anti-CD3 and anti-CD28 0.25 μg/mL (Biolegend) or with phytohemagglutinin (PHA; 0.5 μg/mL; Sigma Aldrich), and co-cultured with or without BMSCs at a ratio of 10:1. After 5 days, the CFSE-labeled PBMCs were harvested, stained with antibodies specific for CD4 and CD8 (anti-CD4-APC; anti-CD8-PE; both Miltenyi), and subjected to analysis with flow cytometry.

For tube formation assay (81), human umbilical vein endothelial cells (HUVECs) were plated in 96-well plates coated with Matrigel (Corning) and co-cultured for 16 h with conditioned culture medium derived from BMSCs or with unconditioned blank control. Bright-field microscopic images of each well were taken for computer-assisted quantification of multiple parameters associated with endothelial network formation, e.g., total master segment length (TMSL/field) (ImageJ 1.51; Bethesda, USA). For the generation of BMSC-conditioned media, the cells were seeded at a density of 1 ×10^4^ cells/cm^2^ in 24-well plates (DMEM + 10%FCS), allowed to adhere overnight, washed once to remove residual protein, and cultured for 24 h with media containing 0.5% FCS to collect the cells secretome. The conditioned a medium was collected and centrifuged to remove cell debris and supernatants, filtered, and stored at −80°C until assayed. Levels of interleukin 6 (IL-6) and vascular endothelial growth factor (VEGF) were assayed by using enzyme-linked immunosorbent assays (ELISA; R&D Systems).

### Exploratory and Descriptive Statistics

Statistical analyses were performed using ANOVA and the Student's *t*-test. All data sets from individual experiments were tested for normal distribution with the Shapiro-Wilks test prior to testing for statistical significance. When performing multiple pair-wise comparisons, one way or two-way ANOVA was used, and Bonferroni *post-hoc* corrections were performed to adjust the *p*-values. For single-group testing, statistical significance was tested by Student's *t*-test (paired or unpaired, two-tailed). If the data did not fit a normal distribution, the Mann-Whitney test or the Wilcoxon matched-pairs test was used (two-tailed confidence intervals, 95%; *p* < 0.05 was considered statistically significant; Prism 5.0; Graphpad Software, USA).

## Results

### Donor Stratification, Phenotypic, and Growth Characteristics of BMSCs

This study included BMSC preparations from 10 adult (38.2 ± 11.1 years) and 13 elderly (72.2 ± 7.5 years) donors, selected upon stratification of clinical background, to dissect the influence of donor age and common comorbidities (Table 2). We found that 60% of the adult cohort and 100% of the elderly cohort presented with comorbidities, with diabetes mellitus in 10% (1/10) and 54% (7/13) of cases (mainly early-stage, 6 non-insulin, and 2 insulin-dependent), respectively, followed by hypertension and other cardiovascular complications.

**Table 2:** Characteristics of bone marrow donors used for isolation of MSCs.

**Donor ID**	**Sex** **(M/F)**	**Age** **(Years)**	**Comorbidities** **(Number)**	**Diabetes mellitus**	**Other types of potential comorbidities diagnosed**
P127	F	16	None	None	None
P264	F	25	None	None	None
P276	F	48	Yes (2)	Yes (NID)	Bone cyst
P285	F	35	None	None	None
P289	F	45	None	None	None
P293	M	48	Yes (1)	None	Hypertension
P308	F	47	Yes (1)	None	Hypertension
P357	M	37	Yes (1)	None	Hyperuraemia
P784	M	33	Yes (1)	None	Hypertension
P819*	F	48	Yes (1)	None	Hypertension
**Adults**	**3 / 7**	**38.2** **±** **11.1**	**6 / 10 (60%)**	**1 / 10 (10%)**	**6 / 10 (60%)**
P237	M	61	Yes (4)	Yes (NID)	Hypertension, HPLA, and RA
P265	M	63	Yes (1)	None	Hypertension
P278	F	82	Yes (2)	None	Hypertension and Hyperuraemia
P316	F	71	Yes (2)	Yes (NID)	Hypertension
P336	M	80	Yes (3)	Yes (NID)	Haematuria and Bradycardia
P354	M	85	Yes (2)	Yes (NID)	Hypertension
P374	M	68	Yes (1)	None	Hypertension
P378	M	78	Yes (1)	None	Hypertension
P386	F	68	Yes (1)	None	Hypertension
P651	F	69	Yes (1)	None	Hypertension
P660	F	65	Yes (2)	Yes (NID)	Hypertension
P777	F	73	Yes (4)	Yes (ID)	Hypertension, CKD3, and HVI
P821*	F	75	Yes (6)	Yes (ID)	Hypertension, CKD3, and HVI/DVT/PE
**Elderly**	**6 / 7**	**72.2** **±** **7.5**	**12 / 12 (100%)**	**7 / 13 (54%)**	**13 / 13 (100%)**

The age range is defined as follows: Adults younger than 50 years, and Elderly older than 60 years, and age values are presented as mean ± SD. The main type of comorbidity studied is diabetes mellitus. (*)Patients 819 and 821 were only included in Figures 5, 6. CKD3, chronic kidney disease grade 3; HLPA, hyperlipoproteinemia; HVI, heart valve insufficiency; DVT, deep-vein thrombosis; PE, pulmonary embolism; RA, rheumatoid polyarthritis; and NID/ID, non-insulin-/insulin-dependent diabetes mellitus.

**Figure 1:**
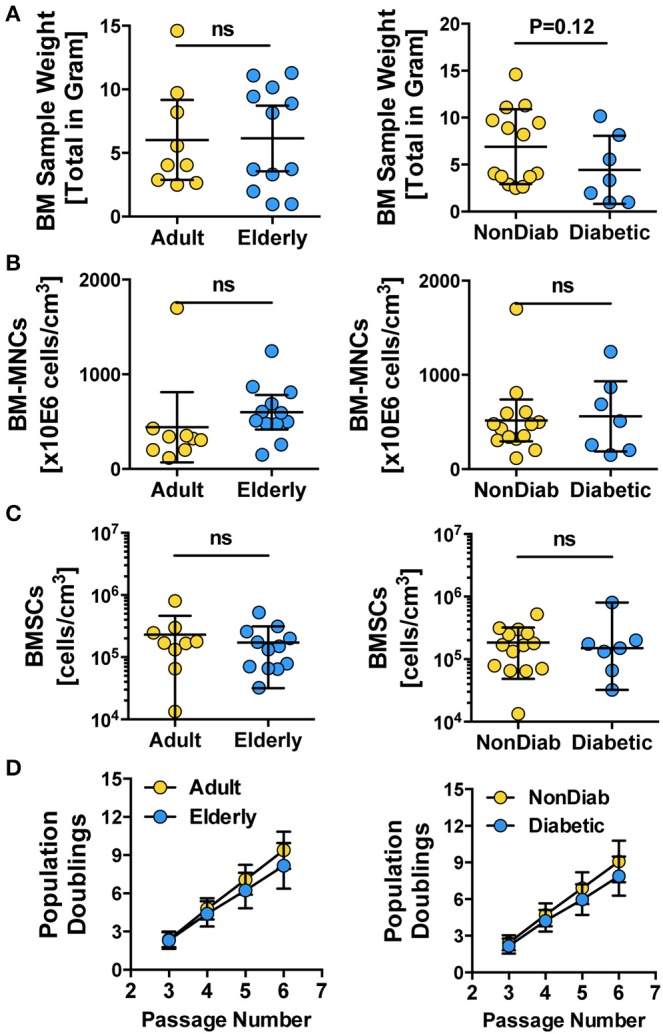
Primary isolation and growth kinetics of BMSCs. **(A–C)** Quality control of BM samples: **(A)** bone marrow sample weight (in grams) and **(B)** number of BM-MNCs per sample (cells/cm^3^), **(C)** number of BMSCs per sample (cells/cm^3^), and **(D)** growth kinetics of BMSCs, with population doublings determined at different passages (P3-6), were quantified for BMSC preparations from adult vs. elderly (*n* = 9 vs. 12) and for non-diabetic vs. predominantly non-insulin-dependent early-stage diabetic donors (*n* = 14 vs. *n* = 7). Data are shown as mean ± SD, and the statistics were evaluated with a Student's *t*-test.

For both the adult and elderly cohort, the BM used as the starting material for cell isolation had a similar sample weight (Figure 1A, left panel), content of BM-MNCs (Figure 1B, left panel), and BMSC frequencies (Figure 1C, left panel). Stratification according to non-diabetic and diabetic donors also resulted in a comparable sample weight (Figure 1A, right panel), number of BM-MNCs (Figure 1B, right panel), and BMSC content (Figure 1C, right panel). Independent of donor age or diabetic status, the isolated BMSCs exhibited similar growth kinetics, as quantified by cumulative population doublings at passages 3 to 6 (Figure 1D). We observed a trend of reduced cell growth with increasing passage number in some of the BMSC preparations generated from the elderly diabetic donors with multiple comorbidities (see below).

**Figure 2:**
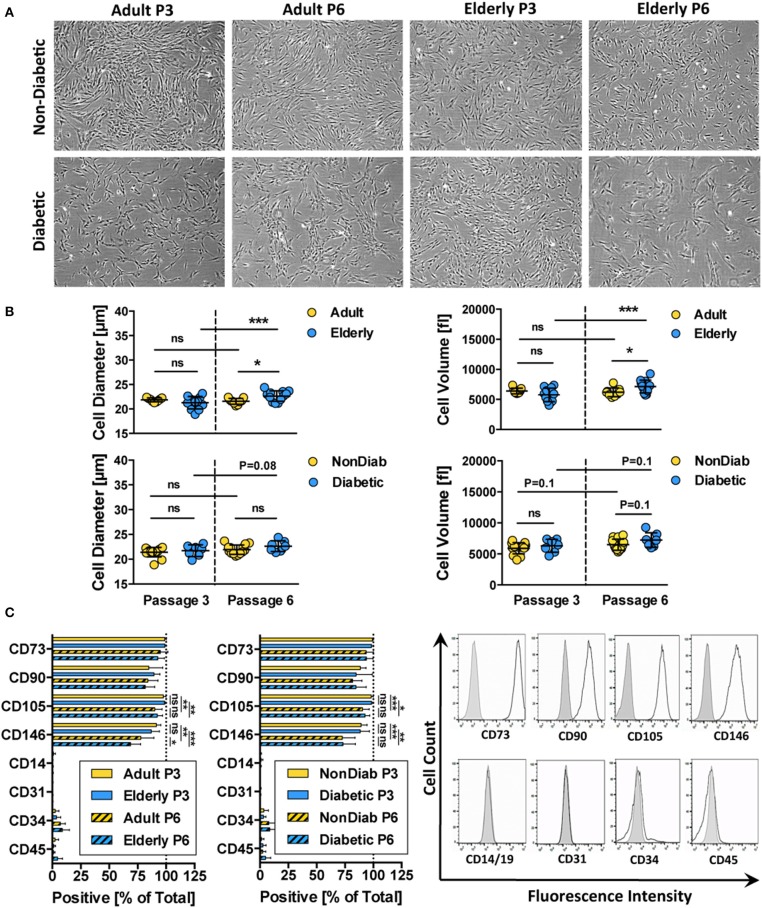
Morphology and immunophenotype of BMSCs. **(A)** Representative bright-field microscopy images of BMSC cultures at passage 3 and 6, comparing adult vs. elderly and non-diabetic vs. diabetic donors, showing typical fibroblast-like morphology with a trend toward more irregular morphology in diabetic donors; **(B)** analysis of cell size and cell volume of trypsin-detached BMSCs from adult vs. elderly (*n* = 9 vs. 12) and non-diabetic vs. diabetic donors (*n* = 14 vs. 7). Adult and elderly have similar cell size and volume at passage 3, and the cell size of adult donor-derived BMSCs does not increase with passages, while elderly donor-derived BMSCs display cell enlargement with increasing passage; and **(C)** flow cytometry analysis of BMSCs (% positive cells; *n* = 6 random adult or elderly donors at early passage 1–3) with representative histograms shown to the right (unlabeled controls are shown in solid gray). The cells highly express typical BMSC-associated markers CD44, CD73, CD90, and CD105 while exhibiting no/weak expression of non-MSC-associated markers CD14, CD31, CD34, and CD45. Data are shown as mean ± SD, and statistical evaluation was performed by Student's *t*-test (*p < 0.05 and ***p < 0.001).

Phenotypic profiling revealed that all of the isolated BMSC cultures had a typical fibroblast-like morphology that was preserved during *in vitro* expansion up to passage 6 (Figure 2A and Figure S1). Regardless of donor age, trypsin-detached spheroid BMSCs had similar cell diameter and volume values at passage 3 (Figure 2B and Figure S1). Cells from adult and elderly donors, however, exhibited an increase in cell diameter and volume with increasing culture time, though this difference only reaches static significance between BMSCs derived from elderly donors at passages 3 and 6 (*p* < 0.001). BMSCs from diabetic donors showed a similar trend of increased cell size and volume, especially at higher passages, but this was not significant (Figure 2B).

Analysis of the cell surface marker pattern revealed that all of the BMSC preparations exhibited a similar surface marker phenotype at passage 3, as defined by the ISCT criteria (69), independent of donor age and disease status (Figure 2C). The isolated cells express typical MSC markers (CD73, CD90, CD105, and CD146) while being negative for contaminating cell populations (CD14, CD19, CD31, CD34, and CD45) such as cells of myeloid, B-cell, endothelial, and hematopoietic origin, respectively.

Interestingly, we noted a weak decline in the expression of CD105 (*p* < 0.05 to *p* < 0.001) and CD146 (*p* < 0.01 to *p* < 0.001) upon extended culture up to P6 in all BMSC preparations, suggesting that long-term culture had a negative impact on the MSC phenotype of the cells. Indeed, a weak decline in CD105 and a stronger decline in CD106- and CD146-expression after extended culture, particularly in DMEM-media, or upon repeated passaging, have been reported previously (82). Functionally, the altered expression of CD105 was found to be associated with decreased osteogenic potential and altered Notch signaling (83).

### *In vitro* Aging, but Not Donor Aging Alters the Transcriptome of Biobanked BMSCs

Next, we studied whether subgroups of BMSCs under resting conditions differed in their gene expression profiles by performing global transcriptome analysis. Multivariate statistical analysis using principal component analysis (PCA) was performed to study the variability between the groups with subsequent visualization of significant differences by hierarchical clustering heat maps and gene-ontology (GO) term enrichment analysis.

**Figure 3:**
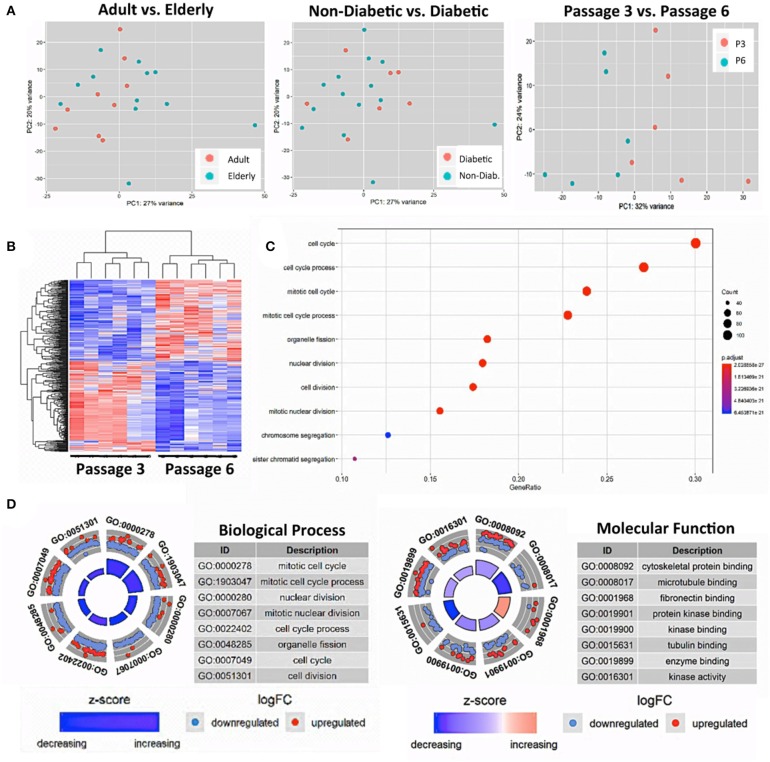
Gene-expression-profiling of BMSCs with RNA-sequencing. **(A)** Principle component analysis (PCA) was employed for the visualization of group separation. Groups were stratified either according to donor age (left panel; *n* = 9 adult vs. *n* = 12 elderly donors), health status (central panel: *n* = 14 non-diabetic vs. *n* = 7 diabetic donors), and passage number (right panel; *n* = 6 random donors at passage 3 vs. 6). The PCA showed clear separation when comparing passage 3 vs. 6 (right panel), but no separation for the comparison of adult vs. elderly or diabetic vs. non-diabetic donors, with a random dot distribution throughout the graph. **(B–D)** passage 3 vs. 6 comparisons: **(B)** hierarchical clustering heat map with expression values sorted according to donor (rows) and gene (columns), where low expression is denoted by blue and high expression by red; **(C)** gene ontology (GO) enrichment analysis, with the “Top 10 Results” for changes in biological process (e.g., cell cycle, nuclear and cell division, and chromosome segregation) shown on the left y-axis and the size of the dots representing the counts of genes involved, while the color of the circle (scaled blue/lowest to red/highest) indicates significance expressed as adjusted *p*-value. **(D)** Combined David and GO “Top 8 Results” database analysis for changes in biological process (left panel; e.g., mitotic cell cycle and nuclear division) and molecular function (right panel; e.g., cytoskeletal protein binding and microtubule binding), with the z-score indicating the overall decrease or increase in expression for certain GO terms and the log-FC analysis indicating the actual number of down- or up-regulated genes within a certain GO term.

Our PCA-analysis found no clear separation between unstimulated BMSCs at P3 either for the comparison of adult and elderly (*n* = 9 vs. *n* = 12 donors) or non-diabetic and diabetic (*n* = 14 vs. *n* = 7 donors) donors (Figure 3A, left and central panel). Our diabetic group consisted of one adult and six elderly donors, and thus the comparison between non-diabetics (8 adults and 6 elderly) and diabetics is mainly a comparison with elderly diabetics (86% of the group), which could potentially weaken this analysis. To clarify this point, we carried out a sub-stratified comparison only between elderly diabetic and elderly non-diabetic donors (*n* = 6 each) (Figure S2). However, this analysis came to the same conclusion as the prior comparison and revealed no apparent differences in transcriptome between the two groups.

This result could suggest either, that the RNA sequencing-obtained gene-expression profiles of our isolated and *in vitro* expanded BMSCs are not affected consistently enough by the parameters age or diabetic status to allow for a robust multivariate statistical separation of these groups under resting conditions or, alternatively, that the studied *in vivo* imprint of the donor (e.g., aging and mild comorbidities) is simply not strong enough or is not maintained after isolation and *in vitro* expansion for several weeks.

Thus, as a positive control, we compared the gene expression pattern of selected donors from the same cohorts at P3 and P6 (*n* = 6 per group). We detected a clear difference between the two groups (Figure 3A, right panel) that confirms earlier reports (16, 38, 54). Our PCA showed a 32% variance in PC1 and 24% variance in PC2, indicating that PC1 (influence of culture time) accounts for most of the observed differences in expression pattern between the two groups. Accordingly, a subsequent hierarchical clustering analysis separated the transcriptome of BMSC preparations by their number of passages into two distinct P3 and P6 groups (Figure 3B).

The “Top 10 Results” from GO-term enrichment analysis of the biological processes predominantly involved identified highly significant changes in gene signatures associated with cell cycle, nuclear cell division, and chromosome segregation (Figure 3C). Concomitant in-depth delineation of the biological process and molecular functions involved using a combined DAVID and GO-database “Top 8 Results” analysis (Figure 3D) revealed that the altered biological processes were associated with changes in cytoskeletal proteins and microtubule binding, tubulin, fibronectin, enzyme, and protein kinase binding and kinase activity.

In summary, we found that the process of BMSC *in vitro* expansion had a strong impact on molecular phenotype, which may mask any rather weak *in vivo* imprint from donor aging and associated mild comorbidities after expansion over several passages in culture, as is typically done for cell production and biobanking. This does not generally exclude potential differences becoming apparent for the assessment of larger age differences (e.g., when comparing very young vs. elderly donors) or when studying the impact of stronger comorbidities.

In order to understand the functional heterogeneity of BMSC preparations, we next analyzed their functional performance in multiple *in vitro* assays typically employed for BMSC characterization (84).

### *In vitro* Aging, but Not Donor Aging Affects BMSC Differentiation Capacity

**Figure 4:**
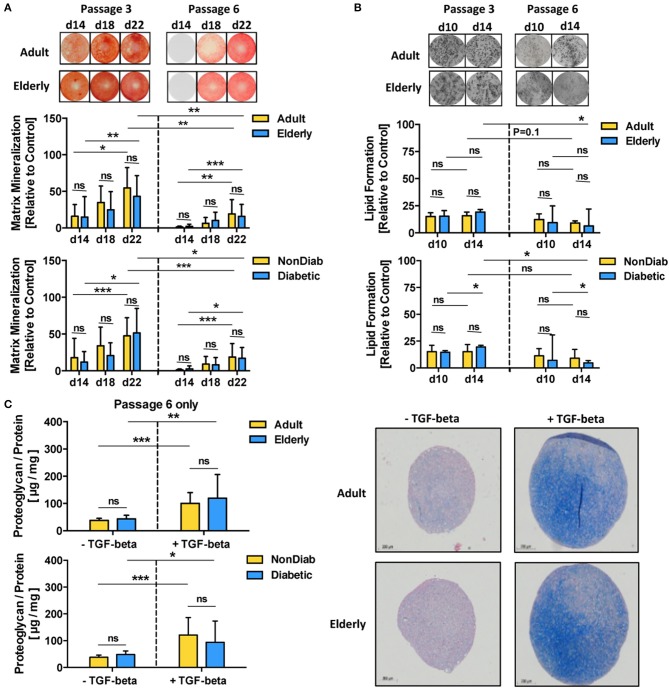
Multilineage differentiation potential of BMSCs. The differentiation capacity of MSCs from adult and elderly (*n* = 9 vs. 12) and non-diabetic and predominantly non-insulin-dependent early-stage diabetic donors (*n* = 14 vs. 7) was assessed with different *in vitro* assays. **(A)** Osteogenic differentiation was assessed by quantification of BMSC matrix mineralization upon osteogenic induction for 14, 18, and 22 days, with representative images for Alizarin red staining shown at the top. Quantification revealed a time-dependent increase in mineralization for all groups (day 14 vs. day 22) both at passages 3 and 6, while there was no difference between adult vs. elderly or non-diabetic vs. diabetic donors at either time point or passage but a strong reduction in mineralization for higher vs. lower passage cells (P6 vs. P3). **(B)** Adipogenic differentiation of BMSCs upon *in vitro* adipogenic induction for 10 and 14 days was quantified by staining of lipid-rich vacuoles with Nile Red, with representative images for vacuole formation shown at the top. Quantification revealed similar vacuole formation for both time points (day 10 vs. day 14), with a trend of modest reduction when comparing higher vs. lower passage cells (P6 vs. P3). There was again no difference between adult vs. elderly or non-diabetic vs. diabetic donors at either time point but a stronger passage-dependent reduction for cells from elderly or diabetic donor donors (P3 vs. P6), though this was less notable for cells from adult or non-diabetic donors. **(C)** Chondrogenic differentiation of BMSCs upon induction with TGF-beta was quantified as the ratio of proteoglycan synthesis relative to protein content, with representative histology images for alcian blue proteoglycan staining of pellet sections shown to the right. There was no difference between BMSCs from adult vs. elderly or non-diabetic vs. diabetic donors. Data are shown as mean ± SD, and statistical evaluation was performed by using a Student's *t*-test or ANOVA followed by post-tests (*p < 0.05, **p < 0.01, and ***p < 0.001).

In accordance with the prior transcriptome analysis setup, we studied the differentiation capacity of BMSCs depending on donor age and diabetic status with adjunct comparison of passages 3 and 6 (Figure 4 and Figures S3, S4). In line with the prior results, we did not find any major differences in the differentiation capacity of BMSC preparations with respect to donor age and the presence of early-stage mild diabetes in the donor cohort under standard culture conditions (Figures 4A–C and Figure S4).

Along with the transcriptome changes observed during replicative *in vitro* aging, we compared the differentiation capacity of BMSC toward osteogenic and adipogenic lineages between P3 and P6 in order to evaluate whether the substantial transcriptional changes may also reflect alterations in functional behavior (Figures 4A,B). Indeed, BMSCs from adult and elderly donors exhibited significantly diminished osteogenic differentiation at passage 6 compared with passage 3 (*p* < 0.01 and *p* < 0.001, Figure 4A), which was also evident for comparison of non-diabetic and diabetic donors at both passages (*p* < 0.001 and *p* < 0.05). In contrast, adipogenic differentiation potential only showed minor changes, mainly reduced lipid formation, when comparing early and late passages or at later readout (*p* < 0.05, Figure 4B). The BMSCs from both adult and elderly donors displayed a similar increase in proteoglycan production upon chondrogenic differentiation with an induction medium containing transforming growth factor-beta (TGF-β) (*p* < 0.01 and *p* < 0.001, Figure 4C). BMSCs from non-diabetic and early-stage diabetic donors displayed similar chondrogenic differentiation capacity upon induction with the specific differentiation medium, thus excluding any major detectable influence of advanced BMSC donor age or diabetic status on chondrogenic differentiation capacity.

We found that assay readout-time had a considerable confounding influence on the obtained results, e.g., when comparing mineralization at day 14 to day 22 for cells at passage 3 and 6 (*p* < 0.05 to *p* < 0.001, Figure 4A) or when studying the optimal time point for the assessment of ALP-activity, which was found to be highest on day 5 for both adult and elderly and both non-diabetic and diabetic cohorts at passage 3 and 6 (Figure S3). This assay-readout time-dependence was less evident for the assessment of adipogenic differentiation (Figure 4B), e.g., when comparing day 10 and 14 at passages 3 and 6. Interestingly, we could detect weakly compromised lipid formation for BMSCs from elderly donors (*p* < 0.05, Figure 4B).

### Cytokine Challenge *in vitro* Reveals Altered Gene Regulation in BMSCs From Select Elderly Donors With Multiple Comorbidities

**Figure 5:**
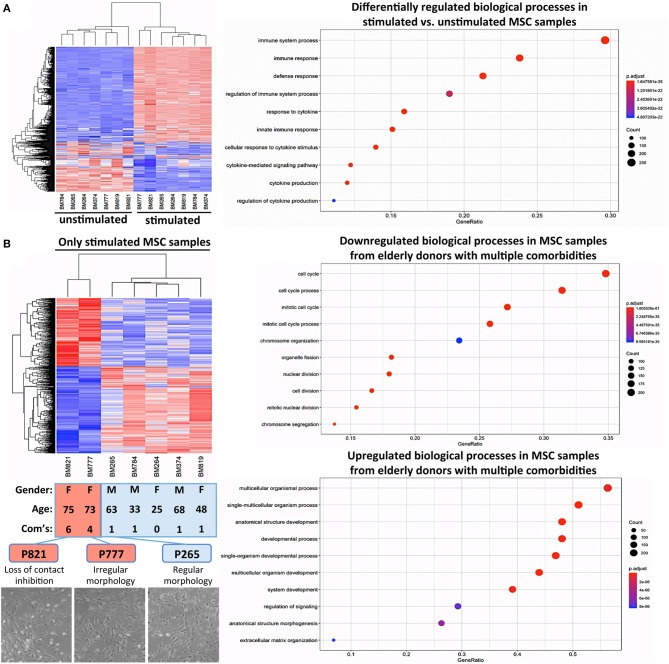
Cytokine challenge reveals altered gene regulation in BMSCs from elderly donors with multiple comorbidities. **(A)** Comparison of unstimulated vs. cytokine-stimulated BMSCs (*n* = 7 donors each): hierarchical clustering heat map with expression values sorted according to donors (rows) and genes (columns), where low expression is denoted by blue and high expression by red, and corresponding gene ontology (GO) enrichment analysis with the “Top 10 Results” for changes in biological process (e.g., immune system process and immune and defense response) shown on the left y-axis, and the size of the dots representing the counts of genes involved, while the color of the circle (scaled blue/lowest to red/highest) indicating significance expressed as adjusted *p*-value. **(B)** Substratified gene expression analysis focusing only on stimulated samples (*n* = 7 donors): BMSCs from elderly donors with multiple comorbidities (P777 and P821, with *n* = 4 and *n* = 6 comorbidities, respectively) cluster separately from both elderly and adult donors with few comorbidities (P265 and P374; and P264, P784, and P819, respectively, *n* = 0–1 comorbidities), indicating that the accumulation of multiple comorbidities during advanced age results in a detectable *in vivo* imprint in the transcriptome of BMSCs. This was accompanied by a decline in cell proliferation and a progression from regular to irregular morphology in culture (representative images at the bottom), resulting in a progressive loss of contact inhibition and cell aggregation. Analysis of the biological processes, showing that P777 and P821 differ under stimulating conditions from the other donors, identified significant downregulation of processes associated with cell proliferation (e.g., mitotic cell cycle, cell division, and chromosome organization), while upregulated processes entailed categories associated with cell differentiation (e.g., multicellular organism process, anatomic structure development, morphogenesis, and ECM organization).

Many clinical applications of BMSCs involve the therapeutic delivery of the cells into challenging *in vivo* environments characterized by inflammation or anoxia (13). Thus, it is indeed advisable to conduct cell-profiling approaches under resting and stimulating conditions (Figure 5A) (85). Stimulation with a cytokine cocktail resulted in a clear separation into two distinct groups in the hierarchical clustering heat map (Figure 5A), with multiple changes in biological processes such as immune system process or immune and inflammatory responses, as typically observed upon cytokine challenge of MSCs, e.g., during potency analysis of MSC products (85, 86).

Substratified expression analysis focusing only on stimulated samples found (Figure 5B), that BMSCs from the two elderly donors with insulin-dependent diabetes and multiple other comorbidities (P777 and P821, *n* = 4 and *n* = 6 comorbidities, respectively) clustered differently from the elderly and adult donors with fewer comorbidities (P265 and P374; and P264, P784, and P819, *n* = 0–1 comorbidities each). However, this has to be interpreted with caution since the number of donors with multiple comorbidities in this analysis was very limited due to their rare occurrence in our biobank. Indeed, changes in BMSC transcriptome upon disease progression to a more advanced stage (e.g., in advanced insulin-dependent diabetes and renal failure) have been reported earlier (55, 58, 60).

In our study, this was associated with a decline in cell proliferation and progression from a regular to a more irregular cell morphology in culture, particularly for BMSCs from elderly donors with insulin-dependent diabetes and multiple comorbidities (Figure 5B, lower panel). Analysis of the biological processes that differ in BMSC specimens from the two elderly donors with multiple comorbidities (Figure 5B, right panel) identified the downregulation of processes associated with cell proliferation (e.g., mitotic cell cycle, cell division, chromosome organization, and organelle fission). In contrast, upregulated processes entailed categories associated with cell differentiation (e.g., multicellular organism process, anatomic structure development and morphogenesis, and ECM organization), thus potentially indicating a progressive loss of the MSC “stem cell” phenotype over time in the presence of multiple strong comorbidities, although any conclusions from this analysis are limited due to the small sample size.

### Inflammatory Challenge Affects BMSC Paracrine Activity

The prior assays involving advanced donor age and the predominant early-stage diabetic status of the included patients showed only minor effects on the *in vitro* expansion and differentiation capacity, as well as the transcriptome of unstimulated BMSCs. The subsequent experiment of *in vitro* cytokine challenge demonstrated a degree of altered responsiveness on the transcriptome level in BMSCs with advanced donor comorbidities.

**Figure 6:**
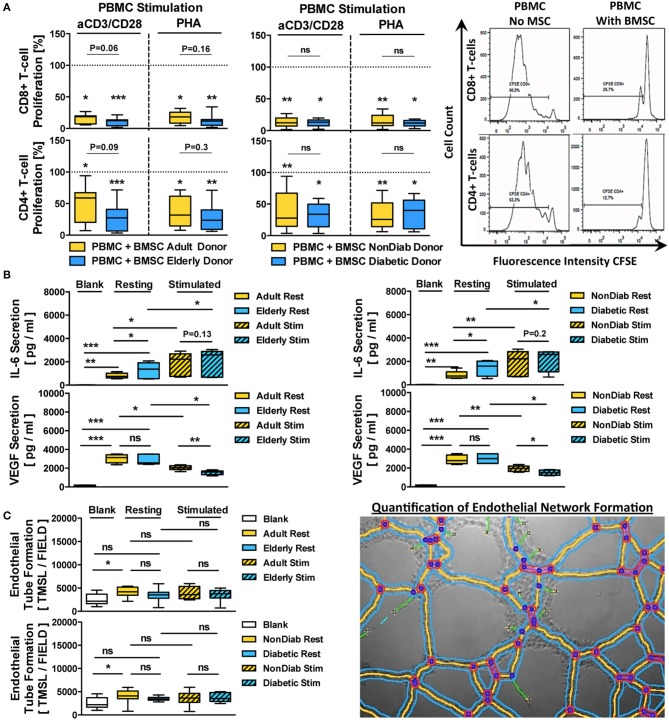
Immunomodulatory and paracrine activity of BMSCs. **(A)** Immunomodulatory activity of BMSCs (*n* = 9 adult vs. *n* = 12 elderly and *n* = 14 non-diabetic vs. *n* = 7 diabetic donors, passage 3) to suppress anti-CD3/CD28- or phytohemagglutinin (PHA)-stimulated peripheral blood mononuclear cell (PBMC)-proliferation. The PBMCs were labeled with the cell proliferation-tracker dye CFSE, activated with either of the two different stimuli, and cocultured for 5 days with BMSCs from adult or elderly donors, in order to assess their capacity to inhibit the proliferation of CD4+ and CD8+ T-cells with flow cytometry, with representative histograms shown to the right. The quantitative assessment of T-cell proliferation is expressed as the percentage proliferation of CD4+ and CD8+ T cells relative to the positive control without BMSCs. Both adult vs. elderly and non-diabetic vs. diabetic donor-derived MSCs are equally potent in inhibiting CD4 and CD8 T-cell proliferation. **(B,C)** Paracrine and proangiogenic activity of BMSCs (*n* = 9 adult vs. *n* = 12 elderly and *n* = 14 non-diabetic vs. *n* = 7 diabetic donors) with and without cytokine stimulation (10 ng/mL of TNF-alpha and IFN-gamma for 24 h): **(B)** secretion of IL-6 and VEGF (pg/mL) in BMSC-conditioned culture media was assessed with ELISA, detecting elevated levels of IL-6 secretion by BMSCs obtained from elderly or diabetic donors, and **(C)** proangiogenic activity of BMSC-conditioned media compared to blank group (medium only) in an endothelial tube formation assay, with a representative image for the quantification of endothelial network formation by quantification of the total master segment length (TMSL/field, with 3–5 images assessed per test condition); TMSL/field was slightly increased for adult and non-diabetic donors. Results are given as box plot ± min-max whiskers. Statistical analysis was performed using either a Student's *t*-test or ANOVA followed by post-tests (*p < 0.05, **p < 0.01, and ***p < 0.001).

In line with these transcriptome changes, we observed more pronounced effects on the secretome and paracrine activity of cytokine-activated BMSCs upon environmental challenge by an inflammatory environment (Figure 6), e.g., upon stimulation with pro-inflammatory cytokines and consecutive readout of angiogenesis in response to their secretome (conditioned media). We found that their immunomodulatory activity to suppress T-cell proliferation was not altered, thus confirming that this is a rather well-preserved process, even if cells are obtained from donors with advanced diabetes and renal failure (55). Nonetheless, the overall impact of these donor parameters on BMSC function was rather modest.

BMSC preparations from both adult and elderly donors and both non-diabetic and diabetic donors effectively suppressed CD8 and CD4 T-cell proliferation in either anti-CD3/CD28 or PHA-stimulated PBMC cultures (Figure 6A). The average remaining proliferation of CD8-responses for adult and elderly BMSCs was 15.9 ± 7.2% and 10.3 ± 6.0% for anti-CD3/CD28-stimulated cultures (*p* < 0.05 and *p* < 0.001) and 17.7 ± 9.0% and 12.1 ± 8.7% for PHA-stimulated cultures compared to control (*p* < 0.05 and *p* < 0.001), with no significant differences between adult and elderly groups for the two different stimuli (*P* = 0.06 and *P* = 0.16, respectively).

The average suppression of CD4-responses was generally weaker, with a remaining proliferation of 46.7 ± 28.7% and 27.9 ± 20.8% for anti-CD3/CD28-stimulated cultures (*p* < 0.05 and *p* < 0.001) and 38.0 ± 23.3% and 27.6 ± 19.8% for PHA-stimulated cultures compared to control (*p* < 0.05 and *p* < 0.001), with no significant differences between adult and elderly groups for the two stimuli (*P* = 0.09 and *P* = 0.3, respectively). A similar suppression pattern of CD8 and CD4 T-cell proliferation in anti-CD3/CD28- or PHA-stimulated co-cultures was found for BMSCs from non-diabetic and diabetic donors (All *p* < 0.01 and *p* < 0.05), while again no significant differences were found between non-diabetic vs. diabetic donors.

Next, we analyzed the secretion of IL-6 and VEGF, two key paracrine mediators associated with BMSC function (Figure 6B). Both, adult and elderly donor-derived BMSCs demonstrated strong secretion of IL-6 compared to the negative control medium (*p* < 0.01 and *p* < 0.001, mean 806 vs. 1,300 pg/mL), with higher IL-6 secretion in BMSC-conditioned media from elderly compared to adult BMSC donors (*p* < 0.05). Similarly, we detected a strong secretion of VEGF by both types of BMSCs compared to negative controls (Both *p* < 0.001, mean 3,033 vs. 2,840 pg/mL), although there was no significant difference between the age groups.

Interestingly, IL-6 and VEGF were inversely regulated upon pro-inflammatory challenge with tumor necrosis factor-alpha (TNF-a) and interferon-gamma (IFN-g), with significant upregulation of IL-6 (*p* < 0.05) but downregulation of VEGF (*p* < 0.05) in stimulated cells compared to resting controls, thus demonstrating an inverse relationship between the two factors under stimulating conditions. Importantly, both BMSCs from adult vs. elderly donors and from non-diabetic vs. diabetic donors showed differential secretion of VEGF upon cytokine stimulation (*p* < 0.01 and *p* < 0.05), which was not the case for IL-6.

When exposing endothelial cells (ECs) to BMSC-conditioned culture medium from adult vs. elderly or non-diabetic vs. diabetic donors (Figure 6C), we found increased proangiogenic activity with media from adult donors compared to negative control (*p* < 0.05, mean 2,551 vs. 4,131 TMSL/field), while conditioned media from elderly donors and the comparison of adult and elderly donors did not reveal any differences. BMSC conditioned medium from non-diabetic donors showed the most profound proangiogenic activity compared to control (*p* < 0.05).

These results taken together indicate a weak but notable influence of advanced donor age and early-stage diabetes on BMSC regulation of its paracrine activity in response to cytokine challenge, while this was less evident for its immunomodulatory activity to suppress T-cell proliferation *in vitro*.

## Discussion

The goal of this study was to understand heterogeneity among BMSC specimens, which are frequently considered for autologous therapy approaches. We asked whether the donor-specific variability in morphological and functional parameters could be explained by intrinsic cell-donor attributes such as variations in donor age and common comorbidities. We thus conducted donor stratification and multi-parameter analysis in a defined patient cohort to allow for a robust readout of individual assay parameters.

Previous studies investigating the impact of donor age and comorbidities on BMSC properties reported partially inconclusive or contradictory outcomes (Table 1). We hypothesized that BMSCs from elderly donors (>60 years), who commonly suffer from mild comorbidities, display reduced regenerative function compared to adult donors (<50 years), who were found to have a much lower burden of common comorbidities. To our surprise, we found that for our prospective stratification, both adult and elderly donors demonstrated on average rather similar performance in most assays and that *in vitro* aging rather than *in vivo* aging and the typically associated mild comorbidities predominantly affected BMSC properties.

### Clinical and Biological Relevance of Donor Aging for BMSC Biobanking Approaches

Many of the treatment indications targeted by allogeneic and autologous BMSC therapies are associated with advanced age, thus making elderly patients with multiple comorbidities one of the high-demand groups for cell-based therapies. They are thus frequently found among the cell donors of our biobank (1). The donor age of BMSC donors is one of the most clearly defined and readily accessible parameters and has been widely investigated, while the impact of certain comorbidities associated with advanced age is more difficult to assess.

Both, autologous and allogeneic BMSC therapies are widely studied, and it is not clear yet which approach will be favored for specific treatment indications (87). Allogeneic approaches provide the great advantage of being able to choose a well-defined starting material, e.g., from developmentally young tissue such as placenta or umbilical cord blood. However, they may come at the cost of immunological incompatibility, which may compromise efficacy and lead to allo-sensitization of the patient. Thus, a major advantage of autologous approaches is their neutral immune profile, but they may be limited by compromised bioactivity of cells sourced from elderly donors with multiple comorbidities, complex diseases, and pharmacological regimens (18, 19, 88).

Many studies focusing on BMSCs in the context of aging have compared various parameters either in cells from younger vs. elderly donors or the impact on cell expansion. These were mainly: (1) Cellular phenotype and proliferation capacity, (2) Gene expression profile, and (3) Various functional parameters, such as mesodermal differentiation capacity and immunosuppressive and paracrine properties. A problem with BMSC characterization is the great number of potentially confounding experimental variables that may impede the readout (85, 89).

### BM-Sample Cellularity, BMSC Growth, Morphology, and Immunophenotype

Multiple studies have reported a decline of BM cellularity (e.g., BM-MNC and BMSC), CFU-F capacity, and growth kinetics with advanced donor age (17, 27, 29–31, 34). BMSCs have been shown to demonstrate a logarithmic decline with increasing age (31, 90), most evidently in the first years of life (e.g., newborns 1/10.000 and teens 1/100.000 BMSCs per BM-MNC), but this was less evident in later life, e.g., when comparing adults vs. elderly donors (e.g., 35-year old 1/250.000, 50-year old 1/400.000, and 60-year old 1/2-million BMSCs per BM-MNC). In our study, we could not detect an age-related difference in cellularity, showing that BM aspirates from adult donors had similar cell content as those from elderly donors (mean 38 vs. 72 years). We could thereby minimize a major confounding sampling bias in the starting material that may have caused initial disparities between the stratified groups.

In prior studies, differences in cell growth were most evident when comparing pediatric vs. elderly donors (27, 34), e.g., defined as the age ranges of 1–5 years vs. 50–70 years, respectively (34). In our study, we found no significant differences in the proliferation of BMSCs from adult and elderly donors, which may be explained by the different time-windows of analysis (Mean 38 vs. 72 years), since the donor population from our biobank does not include pediatric patients and contains few young adults. Our findings are in line with a report by Siegel et al. (33), who also found no correlation between the growth rate of BMSCs and donor age. Nonetheless, we could observe a trend of declining proliferation with successive passages, in particular in BMSCs from elderly donors with multiple comorbidities, although, surprisingly, this was not significant for the entire cohort.

Several reviews have summarized the impact of patient-specific aging and comorbidities on the morphological parameters of BMSCs (18, 19, 91), highlighting cell enlargement, decreased proliferation and replicative quiescence- and senescence-associated ß-galactosidase, as also discussed earlier (26). Siegel et al. reported an impact of donor aging on cell size (BMSCs at P1 from younger donors were smaller) and surface marker expression profile (e.g., increased levels of integrins, PDGFR-beta, and CD90 in younger donors).

In contrast to Siegel et al., who assessed the cell size at passage one, we assessed both cell size and volume of BMSCs at passage three and found that chronological aging had a rather minor influence, which may be due to the longer culture period (two passages longer). Nonetheless, we observed that BMSCs from elderly individuals, but not the younger donors, were more prone to an increase in cell size and volume at later passage (P6). This may support the notion of the so-called “Hayflick limit,” the earlier reaching of proliferative senescence in cells from aged donors that is commonly observed in primary cells (16, 49).

Furthermore, a recent review by Baker et al. suggested that extended *in vitro* cultivation might alter the immunophenotype of BMSCs (17). We analyzed the cellular surface markers proposed by ISCT (69), and found that BMSCs from adult and elderly donors at both passages express typical MSC-associated markers, CD73, CD90, CD105, and CD146 and were negative for CD14, CD31, CD34, and CD45, with a limited influence of passage (decrease of CD105 and CD146). However, we observed that the expression of CD105 and CD146 declines with increasing passage number, suggesting a potential relationship between altered cell surface marker pattern and reduced functional capacity.

Indeed, reduced expression of CD105, CD106, and CD146 after extended culture and repeated passaging, particularly when the cells are cultured in DMEM-media, has been reported previously (82). Surface expression of CD105 can also be affected by the mode of culture (e.g., flasks vs. bioreactor or enzymatic detachment) (92). Downregulation of the TGF-beta receptor endoglin (CD105) and its associated signaling pathways (e.g., the Notch pathway) may also partly explain the evident decline in osteogenic differentiation capacity in BMSCs at increasing passage number (83).

### BMSC Gene Expression Studies

So far, only a small number of studies have comprehensively studied the influence of advanced donor age and comorbidities on the transcriptome and methylome of BMSC products, mainly focusing on the effects of *in vitro* expansion before entering the senescent state (38, 93).

Our PCA of RNA-sequencing-derived gene expression profiles of unstimulated resting BMSC samples at early passage (P3) showed no clear separation between adult vs. elderly or non-diabetic vs. early-stage diabetic donors (both analysis of the whole cohort *n* = 14 vs. 7 or for the sub-stratified analysis of elderly non-diabetics vs. elderly diabetics *n* = 6 each). We compared non-diabetic vs. diabetic donors, since this was the most common and well-defined comorbidity in our cohort and is also frequently studied by others (60).

It should be noted that the majority of our diabetic donors suffered from non-insulin-dependent early-stage type 2 diabetes and that only two of the donors had more advanced insulin-dependent type 2 diabetes and multiple other notable comorbidities, owing to their rare presence in our biobank. However, the two donors with a more advanced disease stage in particular demonstrated differential gene expression upon cytokine challenge, corresponding to gene pathways associated with cellular decline. Davies et al. reported previously that BMSCs from late-stage type 1 diabetic donors show significant alterations in transcriptome compared to healthy controls under resting conditions (55). Indeed, patients who are affected by advanced late-stage type 1 or type 2 diabetes often present with renal failure and other more serious comorbidities and were thus on average sicker than the donors included in our analysis, which may explain the difference (60).

Furthermore, a recent methylome analysis of young and aged adults (*n* = 5 each, mean 22 vs. 75 years, range 20–24 vs. 62–82 years, respectively) at early and late passage (P4 vs. P8) was prospectively designed to distinguish between donor-age- and culture-induced changes (93). The authors found that a larger number of CpGs were differentially methylated in aged donors during culture and biological aging while there were fewer changes in young donors across genic elements, indicating that younger donors are more refractory to culture-induced changes. Furthermore, it was found that the majority of methylation changes appeared to be specific to either young or aged donors, with a subset being specific to long-term culture irrespective of adult donor age.

We also found in our second set of experiments under cytokine-challenge that particular elderly donors with multiple comorbidities (e.g., insulin-dependent diabetes and renal failure) demonstrated differential gene-expression profiles to healthy adults and healthy elderly donors, although these results have to be interpreted with great caution due to the small number of available samples that could be included in this analysis. Analysis of the underlying gene expression pathways indicated that this was associated with a loss of stemness and increased differentiation, going hand in hand with the observed methylome changes in the study above. This may indicate that, while methylome changes between adult and elderly donors are already evident in resting cells, transcriptome changes and their functional impact may become more evident under challenging environmental conditions.

As a positive control, we also analyzed how replicative aging upon *in vitro* expansion influences the expression profiles of BMSCs by comparing the gene expression pattern of six donors at P3 and P6. We found a clear separation between the two groups, thus confirming earlier findings on the matter and demonstrating the validity of our approach (16, 38, 54). Hierarchical clustering heat maps showed distinct gene expression patterns, with a passage-dependent decline in the expression of genes associated with cell cycle and cell proliferation. Indeed, earlier studies have demonstrated continuous and progressive gene-expression changes in BMSCs upon long-term culture expansion (16, 38). When comparing BMSCs at culture intervals from P2 up to P11, gene expression changes accumulated with each additional passage, with P2-3 vs. P4-5 vs. P6-11 being distinguishable, demonstrating the great analytic power of the method.

Similarly, a recent study documented accumulating transcriptome drift in UC-MSCs cultured until replicative senescence, with transcriptome changes becoming evident at P5, with a greater increase when reaching senescence at P9-12 (54), thus making it possible to distinguish between early passage (P2-4), medium passage (P6-8), pre-senescent (P10-12), and senescent (P14) cells. Both of these examples clearly illustrated that *in vitro* expansion influences BMSC gene expression signatures, which can dilute or mask any consistent *in vivo* signatures associated with donor aging and comorbidities.

### Functional Assessment: Differentiation, Paracrine, and Immunomodulatory Activity

Since the key report by Pittenger et al. (84), the majority of studies on BMSCs have assessed multilineage differentiation potential as part of the minimal criteria for their characterization (69). Importantly, those that have studied the impact of aging and comorbidities often reported a negative impact of advanced donor age, with differential effects on the individual lineages (e.g., osteogenic, chondrogenic, and adipogenic potential). A review by Baker et al. pointed out that this is disputable and that approximately half of the studies do not find differences (17).

Multiple reports have shown no effect or that osteogenic differentiation decreases with increasing donor age (26, 29, 31, 33, 94). D'Ippolito et al. found a reduced ALP-positive CFU-F number and osteogenic potential in younger vs. elderly donors (3–36 vs. 41–70 years, respectively) (31). Müller et al. also reported a strong donor-age related decline in the osteogenic potential of BMSCs isolated from total hip arthroplasty patients ( ≤ 50 years 7/11 or 63% positive, ≥60 years 5/19 or 26% positive) (94).

Furthermore, Stolzing et al. reported that osteogenic and chondrogenic potential were diminished with advanced age, while adipogenic differentiation was not (29). However, others found no age-dependent differences in differentiation capacity for either lineage (24, 33, 45). This inconsistency between study results may be explained by differences in methodology, e.g., using cells at different passages (26), as also indicated by our transcriptome analysis.

We thus analyzed the differentiation potential of adult vs. elderly and non-diabetic vs. early-stage diabetic donor-derived BMSCs at both early and late passage (P3 vs. P6). We found that the differentiation of BMSCs toward osteogenic, adipogenic, and chondrogenic lineages was mainly independent of the age and mild comorbidities of the donor and that *in vitro* aging, rather than *in vivo* aging, had a notable impact.

A large share of the therapeutic activity of BMSCs is attributed to their secretion of trophic and immunomodulatory factors (95, 96), which can be modulated by the environment the cells persist in or are brought into. Several reviews have summarized how these properties are potentially altered in the context of donor aging and its associated comorbidities (18, 21, 97).

Siegel et al. reported increased expression of IL-6 by BMSCs in association with aging (33), while Efimenko et al. reported reduced expression of VEGF and the loss of angiogenic potential in elderly donors with cardiovascular complications who more frequently presented with diabetes. We also found that BMSCs from elderly donors produced higher levels of IL-6, while both elderly and diabetic donors showed a stronger decline in VEGF-production under stimulating conditions, which was also reflected in lower *in vitro* angiogenic activity.

Considering their immunomodulatory activity, Siegel et al. did not find a correlation between increased donor age and a loss of immunomodulatory activity in BMSCs in a large cohort of more than 50 donors aged 13–80 years (33). Furthermore, two studies did not find a negative impact of diabetes when comparing BMSCs from healthy donors either to early- and late-stage type 1 diabetic donors (55) or to newly diagnosed type 1 diabetics (56). In contrast, Manchini et al. (*n* = 27 adult vs. *n* = 23 elderly donors, cut-off 65 years) and Serena et al. (*n* = 4 donors each) reported a negative impact of age, atherosclerosis, obesity and type 2 diabetes on the immunomodulatory properties of adipose-derived MSCs.

Similarly to Siegel, Davies, and Yaochite and colleagues, we did not observe any differences in the capacity of BMSC to suppress CD4 or CD8 T-cell proliferation in anti-CD3/CD28- or PHA-stimulated cultures with respect to age and comorbidities. This may be explained by the rather weak comorbidities in our patient cohort and the different age cut-offs for stratification of the groups or by differences in methodology.

## Study Limitations

Qualifying adult stem cell sources in biobanking approaches is of major importance for understanding their behavior in preclinical and clinical studies (1). Importantly, the results obtained in most of the studies by other groups and also our own studies are shaped by the starting material, cell isolation and expansion protocols, and consecutive analysis methods. In the following sections, we discuss a few prominent limitations that are important for the interpretation of the results of this and other studies.

### Starting Material and Baseline Characteristics of Diabetic Patients

Different outcomes considering an *in vivo* imprint of the cell donor and tissue source may be obtained when using different starting materials (e.g., adipose or placental tissue instead of BM) (98). We have predominantly banked BM-MSCs at our facility so far, and we can thus not extend our analysis to MSCs derived from other tissue sources in the same depth. Our study is limited by the absence of control cells from young and healthy volunteer donors from a commercially available source, e.g., American Type Culture Collection (ATCC). However, multiple MSC batches from young, healthy donors isolated at our facility were included, and we here focused on the clinically relevant patient population, who are most in need of autologous BMSC-based therapies. Another important aspect is the baseline characteristics of the patients and their comorbidities, in particular the elderly diabetic patients. As discussed above, the majority of the diabetic donors were non-insulin-dependent early-stage diabetic (where few differences were found), while two elderly donors with more progressed insulin-dependent type 2 diabetes and multiple comorbidities demonstrated more substantial phenotypic and functional alterations in line with results from other groups (60), which should be anticipated when interpreting the results for clinical use of BMSCs.

### Cell Isolation, Expansion, and Enzymatic Detachment Protocols

Our protocol is based on one of the most commonly used methods, separation of the BM-MNCs with density-gradient centrifugation and plastic adherence, with culture and expansion employing DMEM-LG containing 10% FCS. It is noteworthy that alternative culture media (e.g., chemically defined serum-/xeno-free MesenCult-XF medium or StemPro XF and SF media) and growth supplementation (e.g., MSCGM SingleQuots instead of 10% FCS) have become popular and may yield different results when considering *in vitro* aging and donor-specific *in vivo* imprint. Furthermore, our cell detachment and passaging protocol is based on the most commonly used protease trypsin, but other protocols (e.g., employing more gentle cell dissociation with dispase instead of trypsin), may lead to different observations in terms of *in vitro* aging, while it is rather unlikely that any *in vivo* donor imprint is better preserved by using alternative enzymatic detachment.

### Transcriptome Analysis Method

Gene regulation at the post-transcriptional level is of great importance in (adult) stem cells, and differences in transcriptome or the lack thereof have to be interpreted with caution and should not be equaled with proteome or functional conclusions. We thus paralleled our transcriptome analysis with multiple functional *in vitro* assays, partly under challenging inflammatory conditions, to mimic the cells' responsiveness in a disease context (85, 86). We also used the known *in vitro* aging effect during successive passaging as a positive control to align transcriptional with functional outcomes. Novel methods such as epigenetic methylome analysis and Ribo-profiling are of great interest (93, 99). The latter attempts to better reflect the “active” proteome by employing analysis of mRNA associated with polysomes, which may more closely reflect the true levels of the actively translated transcripts in the cells, although the method is technically more challenging than the already established RNAseq (99).

## Summary and Conclusion

Our study demonstrates that donor age and its typically associated mild comorbidities may exert less influence on the phenotype and functionality of BMSCs than previously assumed and that these two parameters do not explain the inherent donor variation in our biobank. In fact, *in vitro* aging rather than *in vivo* aging exerted a strong influence on the cellular properties in our setting, with prolonged culture expansion impairing the regenerative functions of BMSCs at later passages, which should therefore be strongly controlled for in preclinical and clinical studies. Therapeutic approaches would best require a large number of minimally expanded cells with optimum potency. Since sufficient BMSC numbers can only be obtained by extensive expansion, this might be a limiting factor for using BMSCs in cellular therapy, unless a sufficient amount of starting material allowing for limited expansion can be obtained. Alternative tissue sources with better expansion capacity (e.g., perinatal tissue sources such as placenta or umbilical cord), may thus offer certain advantages, but, similarly to other MSC sources, bear an additional risk of thromboembolic complications when applied systemically (4). Therefore, the ideal source of therapeutic MSCs still needs to be defined, and therapeutic approaches utilizing BMSCs should critically review *in vitro* expansion protocols.

## Data Availability Statement

The datasets generated for this study can be found in Gene Expression Omnibus, The expression raw data will be available at Gene Expression Omnibus upon publication of this manuscript. GEO-Accession-ID: GSE139073 and are available at https://www.ncbi.nlm.nih.gov/geo/query/acc.cgi?acc=GSE139073.

## Ethics Statement

BMSCs were received from the Core-Facility “Cell Harvesting” of the BIH Center for Regenerative Therapies (BCRT). The cells were isolated from metaphyseal bone marrow (BM) biopsies from patients undergoing hip replacement at Charité University Hospital, as previously stated (1, 66–68). Written informed consent was given, and ethics approval was obtained from the local ethics committee/institutional review board (IRB) of the Charité University Hospital.

## Author Contributions

GM, SG, and AA: conception and design, administrative support, data analysis and interpretation. GM, SG, GD, PR, DS, and TZ: financial support. GM, SG, AA, RC, JS, TQ, FS, DJ, AB, SRe, DK, MS, SS, SRi, NS, CB, JK-M, UK, TZ, and KJ: collection and assembly of data. GM, SG, DS, and AA: manuscript writing. AA, RC, JS, TQ, FS, DJ, AB, SRe, DK, MS, SS, SRi, NS, CB, JK-M, UK, TZ, KJ, DS, PR, GD, GM, and SG: final approval of manuscript.

### Conflict of Interest

The authors declare that the research was conducted in the absence of any commercial or financial relationships that could be construed as a potential conflict of interest.

## Supplementary Material

Figure S1 Morphological heterogeneity in BMSC preparations from individual adult and elderly donors. Representative bright-field microscopy images (40x magnification) of *n* = 21 BMSC preparations at passages 2–3 vs. 5–6, generated either from adult (*n* = 9) or elderly donors (*n* = 12) with typical fibroblast-like morphology and some minor phenotypic variation being evident in both cohorts. The BMSCs derived from elderly donors with diabetes mellitus [DIAB; Elderly P6; e.g., P237, P316, P336, P354, P660, and P777)] in particular appear to show a trend of deterioration in morphology and slower growth at higher passages, which also appears to be the case for the one diabetic donor in the adult group (Adult P6; P276).

Figure S2 Focused gene-expression profiling of biobanked resting BMSCs from elderly donors with or without predominantly non-insulin-dependent early-stage diabetes**. (A,B)** Principle component analysis (PCA) was employed to study group separation comparing elderly donors with or without diabetes (*n* = 6 each), with a random dot distribution throughout the graph.

Figure S3 Heterogeneity in osteogenic and adipogenic differentiation potential for BMSCs from individual donors. **(A)** Osteogenic differentiation: Alizarin red staining of BMSC matrix mineralization for individual donors (*n* = 9 adult vs. *n* = 12 elderly donors) at passages 3 and 6 upon *in vitro* osteogenic induction for 14, 18, and 21 days. **(B)** Adipogenic differentiation: Nile Red staining of lipid-rich vacuoles for BMSCs from individual donors (*n* = 9 adult vs. *n* = 12 elderly donors) at passages 3 and 6 upon *in vitro* adipogenic induction for 10 and 14 days. In general, both adult and elderly BMSCs display a very large time-dependent heterogeneity in osteogenic and adipogenic differentiation, which makes any predictions of functional performance difficult.

Figure S4 ALP-activity during osteogenic differentiation of adult vs. elderly and non-diabetic vs. diabetic donors. Alkaline phosphatase (ALP) enzyme activity was assessed upon *in vitro* osteogenic induction of BMSCs for 0, 5, and 10 days at P3 and P6 either for: **(A)** Adult vs. elderly donors (*n* = 9 and *n* = 12) or **(B)** non-diabetic vs. diabetic donors (*n* = 14 vs. *n* = 7, respectively). For all comparisons, ALP activity peaks at day 5, with similar values at P3 and P6. Data are shown as mean ± SD and statistical evaluation was performed by using a Student's *t*-test or ANOVA followed by post-tests (^*^*p* < 0.05, ^**^*p* < 0.01, and ^***^*p* < 0.001).
