# Supplementary figures and images for: Multi-Parameter Analysis of Biobanked Human Bone Marrow Stromal Cells Shows Little Influence for Donor Age and Mild Comorbidities on Phenotypic and Functional Properties
Source: Front Immunol. 2019 Nov 8;10:2474. doi: 10.3389/fimmu.2019.02474 (PMC6857652; doi:10.3389/fimmu.2019.02474)

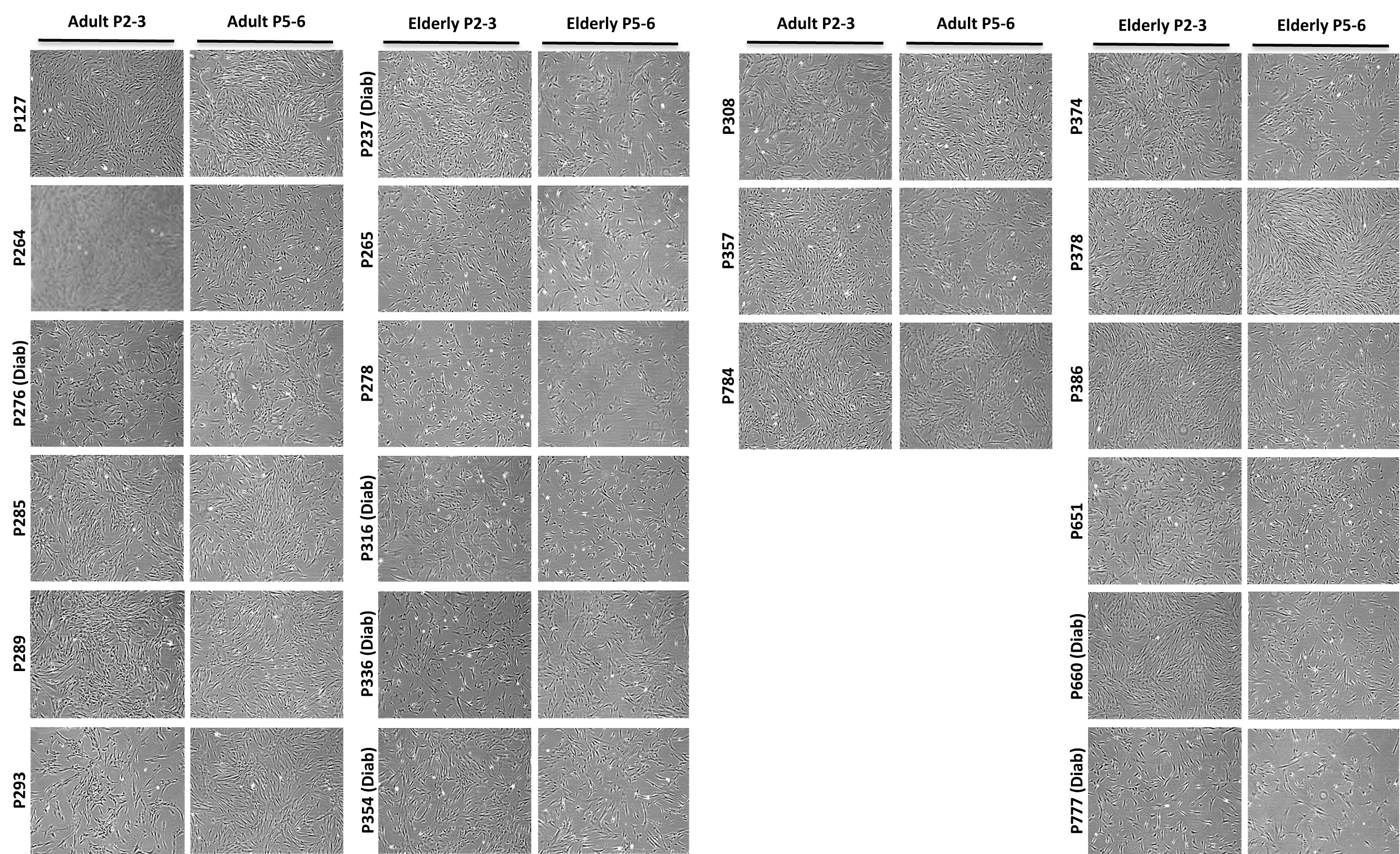

Supplement: Figure S1 — Morphological heterogeneity in BMSC preparations from individual adult and elderly donors. Representative bright-field microscopy images (40x magnification) of n = 21 BMSC preparations at passages 2–3 vs. 5–6, generated either from adult (n = 9) or elderly donors (n = 12) with typical fibroblast-like morphology and some minor phenotypic variation being evident in both cohorts. The BMSCs derived from elderly donors with diabetes mellitus [DIAB; Elderly P6; e.g., P237, P316, P336, P354, P660, and P777)] in particular appear to show a trend of deterioration in morphology and slower growth at higher passages, which also appears to be the case for the one diabetic donor in the adult group (Adult P6; P276). [file Image_1.tif]

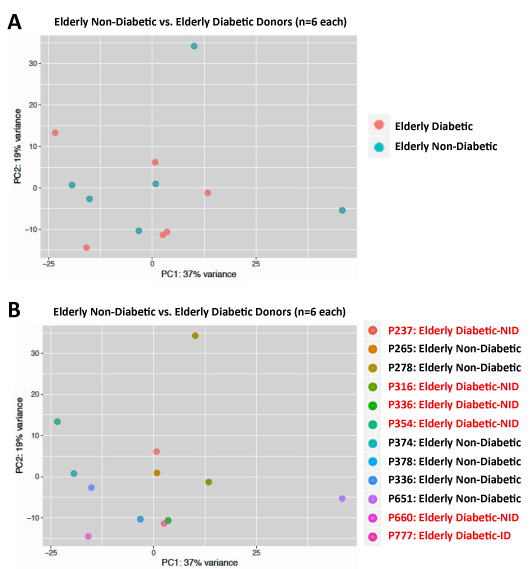

Supplement: Figure S2 — Focused gene-expression profiling of biobanked resting BMSCs from elderly donors with or without predominantly non-insulin-dependent early-stage diabetes. (A,B) Principle component analysis (PCA) was employed to study group separation comparing elderly donors with or without diabetes (n = 6 each), with a random dot distribution throughout the graph. [file Image_2.tif]

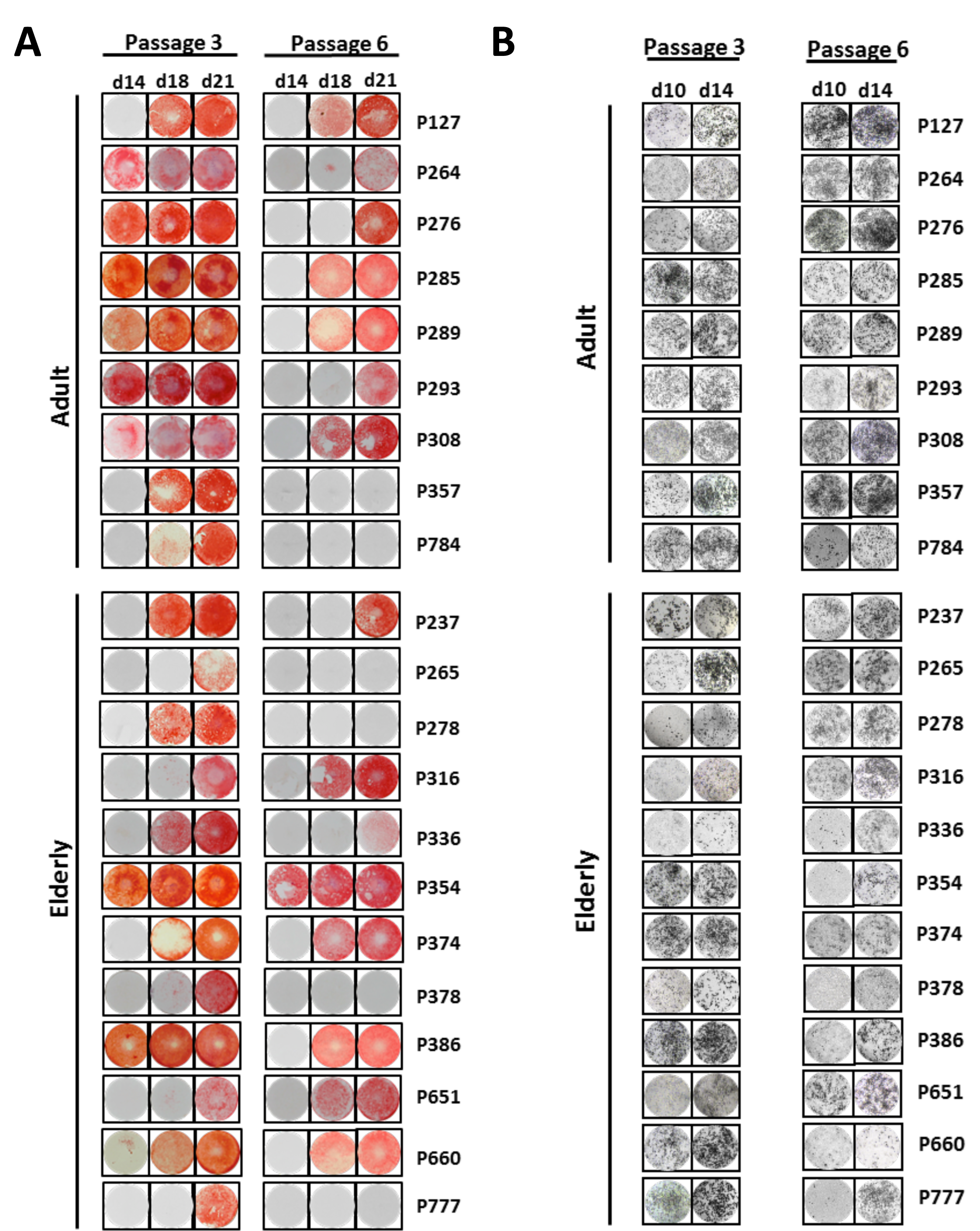

Supplement: Figure S3 — Heterogeneity in osteogenic and adipogenic differentiation potential for BMSCs from individual donors. (A) Osteogenic differentiation: Alizarin red staining of BMSC matrix mineralization for individual donors (n = 9 adult vs. n = 12 elderly donors) at passages 3 and 6 upon in vitro osteogenic induction for 14, 18, and 21 days. (B) Adipogenic differentiation: Nile Red staining of lipid-rich vacuoles for BMSCs from individual donors (n = 9 adult vs. n = 12 elderly donors) at passages 3 and 6 upon in vitro adipogenic induction for 10 and 14 days. In general, both adult and elderly BMSCs display a very large time-dependent heterogeneity in osteogenic and adipogenic differentiation, which makes any predictions of functional performance difficult. [file Image_3.tif]

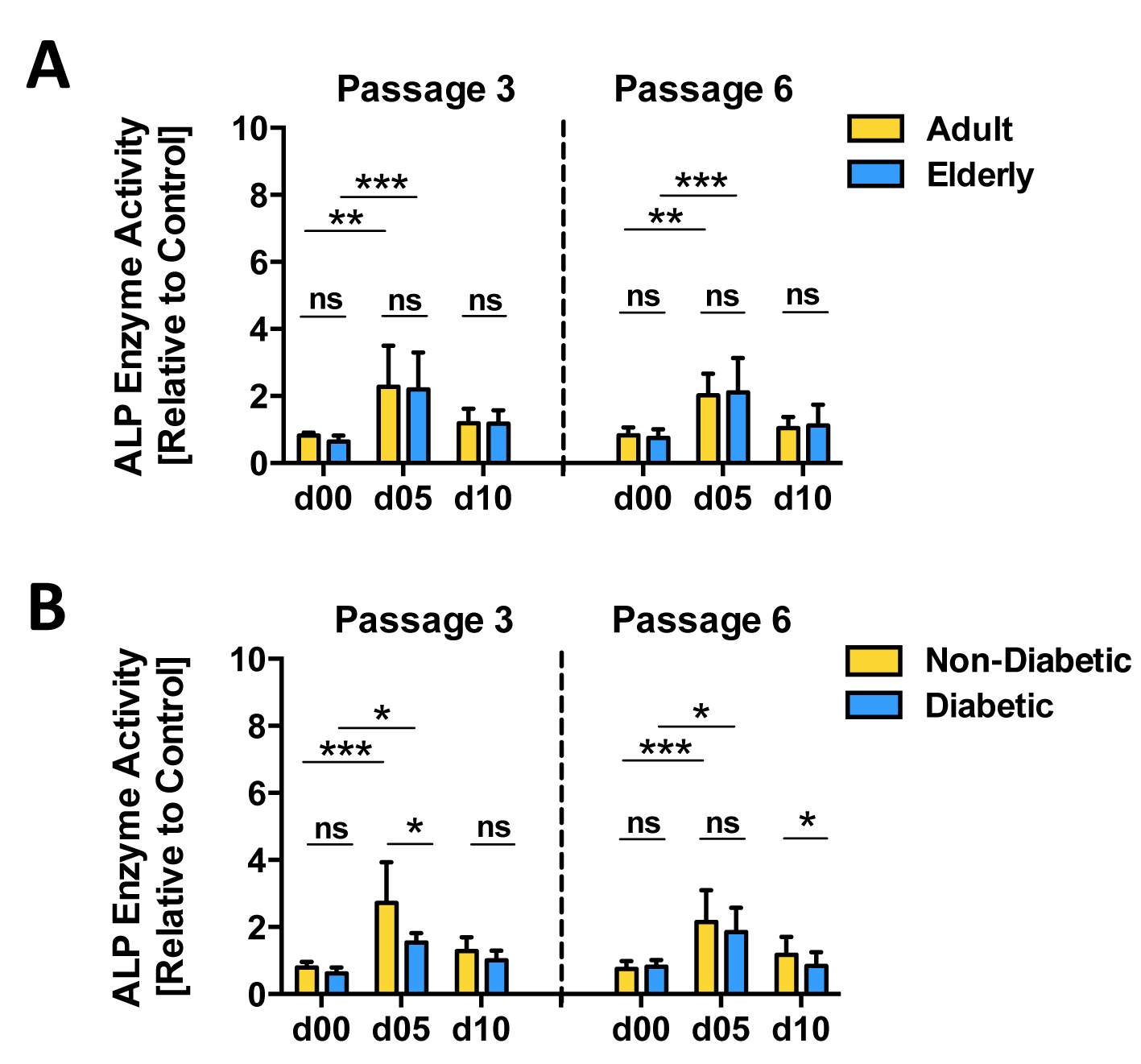

Supplement: Figure S4 — ALP-activity during osteogenic differentiation of adult vs. elderly and non-diabetic vs. diabetic donors. Alkaline phosphatase (ALP) enzyme activity was assessed upon in vitro osteogenic induction of BMSCs for 0, 5, and 10 days at P3 and P6 either for: (A) Adult vs. elderly donors (n = 9 and n = 12) or (B) non-diabetic vs. diabetic donors (n = 14 vs. n = 7, respectively). For all comparisons, ALP activity peaks at day 5, with similar values at P3 and P6. Data are shown as mean ± SD and statistical evaluation was performed by using a Student's t-test or ANOVA followed by post-tests (*p < 0.05, **p < 0.01, and ***p < 0.001). [file Image_4.tif]
